# Mitochondrial genome characterization and phylogenetic analysis of arbuscular mycorrhizal fungus *Rhizophagus* sp

**DOI:** 10.1080/23802359.2020.1715868

**Published:** 2020-01-24

**Authors:** Xu Wang, Mingdao Wang, Xinyu Liu, Ailing Tan, Na Liu

**Affiliations:** College of Life Sciences, Henan Agricultural University, Zhengzhou, Henan, China

**Keywords:** *Rhizophagus*, mitochondrial genome, phylogenetic analysis

## Abstract

In the present study, the complete mitochondrial genome of *Rhizophagus* sp. was assembled by the next-generation sequencing. We found that the complete mitochondrial genome of *Rhizophagus* sp. is 50,449 bp in length and consists of 14,741 (29.22%) adenine, 9427 (18.69%) cytosine, 9248 (18.33%) guanosine, and 17,033 (33.76%) thymine. The genome contains 24 conserved core protein-coding genes, 25 tRNA genes, and 2 rRNA genes. Phylogenetic analysis based on the combined mitochondrial gene set showed that *Rhizophagus* sp. has a close relationship with *Rhizophagus fasciculatus*, *Glomus irregular*, and *G. intratadices.*

Arbuscular mycorrhizal fungi (AMF), belonging to the Glomeromycotina, form mutualistic symbioses with about 80% of land plants (Spatafora et al. 2016). In this symbiosis, AMF help plants obtain nutrients such as phosphorus and nitrogen (Bonfante and Genre 2010). In return, plants provide lipids and sugars for fungi (Wang et al. 2017). In addition, arbuscular mycorrhizal fungi can enhance the tolerance of plants to biotic and abiotic stresses (Strack et al. 2003). The genus *Rhizophagus* is a group of important arbuscular mycorrhizal fungi, which plays an important role in promoting plant growth and natural carbon and nitrogen cycle (Le Pioufle et al. 2019). The study of the mitochondrial genome of *Rhizophagus* species will help us to understand the evolution and phylogeny of *Rhizophagus* species.

The specimen *Rhizophagus* sp. was collected from Zhengzhou, Henan, China (113°20′E; 34°26′N) and was stored in Henan Agricultural University (No. Rsp009). Total genomic DNA was extracted using a Fungal DNA Kit D3390-00 (Omega Bio-Tek, Norcross, GA, USA), and stored in the sequencing company (BGI Tech, Shenzhen, China). Sequencing libraries were constructed using a NEB Next Ultra II DNA Library Prep Kit (NEB, Beijing, China) following the manufacturer’s instructions. Whole genomic sequencing was performed using an Illumina HiSeq 2500 Platform (Illumina, San Diego, CA, USA). The *Rhizophagus* sp. mitochondrial genome was assembled and annotated according to the previous described methods (Li, Liao, et al. 2018; Li, Wang, et al. 2018; Li et al. 2019a, 2019b).

The complete mitochondrial genome of *Rhizophagus* sp. is 50,449 bp in length and consists of 14,741 (29.22%) adenine, 9427 (18.69%) cytosine, 9248 (18.33%) guanosine, and 17,033 (33.76%) thymine. The genome contains 24 conserved core protein-coding genes, 25 tRNA genes, and 2 rRNA genes. The *Rhizophagus* sp. mitochondrial genome sequence was submitted to GenBank under the accession number of MN878008.

Bayesian inference (BI) was used to create a phylogenic tree based on the combined mitochondrial gene set according to previously described methods (Li, Yang, et al. 2018; Li et al. 2019c) (Figure 1). Bayesian analyses were performed with MrBayes v3.2.6 (Ronquist et al. 2012). Phylogenetic analysis showed that *Rhizophagus* sp. has a close relationship with *Rhizophagus fasciculatus*, *Glomus irregular*, and *G. intratadices* (Lee and Young 2009).

**Figure 1.**
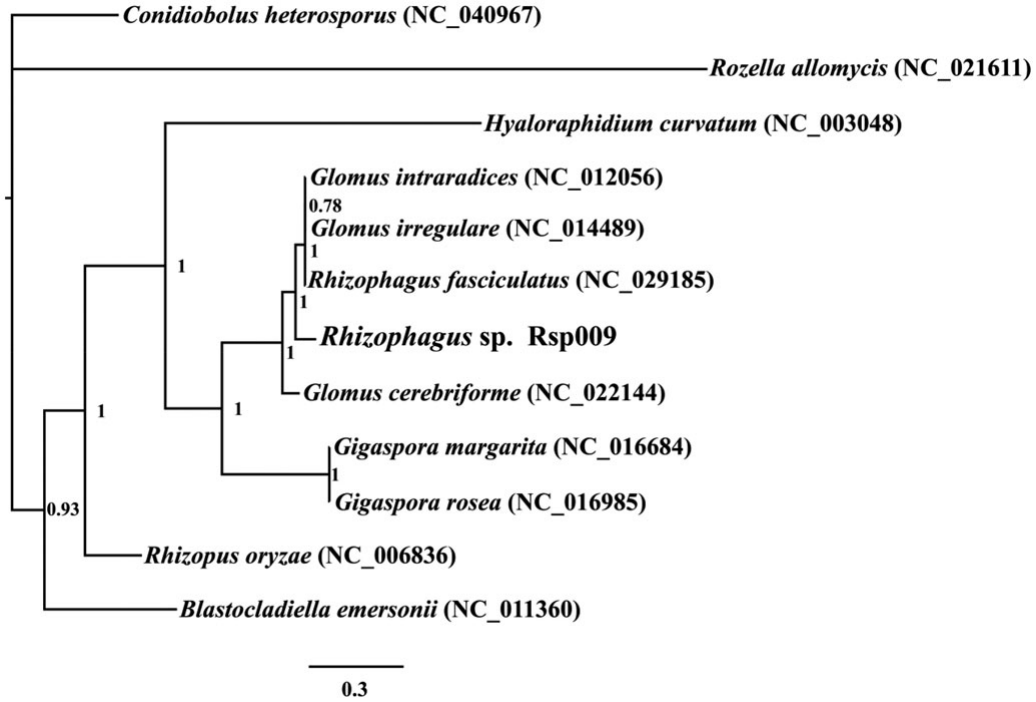
Phylogenetic relationships of 12 species based on Bayesian inference analysis of 14 conserved protein-coding genes. Support values are Bayesian posterior probabilities. The brackets after the species name are GenBank accession numbers of species used in the phylogenetic analysis.
